# Immunomodulation by intravenous omega‐3 fatty acid treatment in older subjects hospitalized for COVID‐19: A single‐blind randomized controlled trial

**DOI:** 10.1002/ctm2.895

**Published:** 2022-09-19

**Authors:** Hildur Arnardottir, Sven‐Christian Pawelzik, Philip Sarajlic, Alessandro Quaranta, Johan Kolmert, Dorota Religa, Craig E. Wheelock, Magnus Bäck

**Affiliations:** ^1^ Department of Medicine Solna Karolinska Institutet Theme Heart, Vessels, and Neuro Karolinska University Hospital Stockholm Sweden; ^2^ Division of Physiological Chemistry 2 Department of Medical Biochemistry and Biophysics Karolinska Institute Stockholm Sweden; ^3^ The Institute of Environmental Medicine Karolinska Institutet Stockholm Sweden; ^4^ Department of Neurobiology Karolinska Institutet and Theme Ageing Karolinska University Hospital Stockholm Sweden; ^5^ Department of Respiratory Medicine and Allergy Karolinska University Hospital Stockholm Sweden

Dear Editor,

COVID‐Omega‐F (“Resolving inflammatory storm in COVID‐19 patients by Omega‐3 Polyunsaturated fatty acids”) is a randomized controlled trial on intravenous (i.v.) omega‐3 (*n*‐3) polyunstaurated fatty acids (PUFA) treatment of coronavirus disease 2019 (COVID‐19).^1^ We here report that COVID‐Omega‐F met its primary endpoint of changes in inflammatory biomarkers for white blood cell counts and lipid metabolites, and we also present exploratory studies of the underlying mechanisms.

A disturbed balance in endogenous PUFA metabolism increases proinflammatory and leukotoxic lipid mediators with concomitant decrease in lipid mediators for the resolution of inflammation. The *n*‐3 PUFA eicosapentaenoic acid (EPA) and docosahexaenoic acid (DHA) decrease C‐reactive protein (CRP) in COVID‐19.^2^, ^3^ I.v. n‐3 PUFA reduces hyperinflammation in other critical infectious conditions^4^ but has not previously been studied in COVID‐19, and the mechanisms involved remain unknown.

Patients with COVID‐19 requiring hospitalization were randomized 1:1 to a once daily i.v. infusion (2 mL/kg) of either placebo (0.9% NaCl) or n‐3 PUFA emulsion containing 10 g of fish oil per 100 mL for 5 days. The full study protocol (clinicaltrials.gov/NCT04647604) has been published^1^ and is detailed in Supplementary Methods. The study was completed for 22 older subjects (mean age 81 ± 6.1 years; Supplementary Table 1, Supplementary Fig 1) before COVID‐19 vaccination was available and during the introduction of cortisone treatment of COVID‐19, which was equally distributed within the placebo (n = 7) and active treatment groups (n = 7). One patient in the placebo group received remdesivir, and no patients received convalescent plasma or any other specific anti‐viral treatments.

The uncontrolled inflammatory response in COVID‐19 is characterized by a high neutrophil to lymphocyte ratio (NLR), which was significantly decreased by n‐3 PUFA (Figure 1). Lymphocyte count was increased 0.4 ± 0.08 × 10^9^/L for n‐3 PUFA vs. 0.03 ± 0.16 × 10^9^/L for placebo (P = 0.03). At the early time point, CRP was significantly lower 24–48h after n‐3 PUFA compared with placebo treatment. Plasma levels of CRP and the cytokines interleukin (IL)‐1β, IL‐6, and TNF‐α were not significantly different between the groups at the end of the study (Supplementary Fig 2).

**FIGURE 1:**
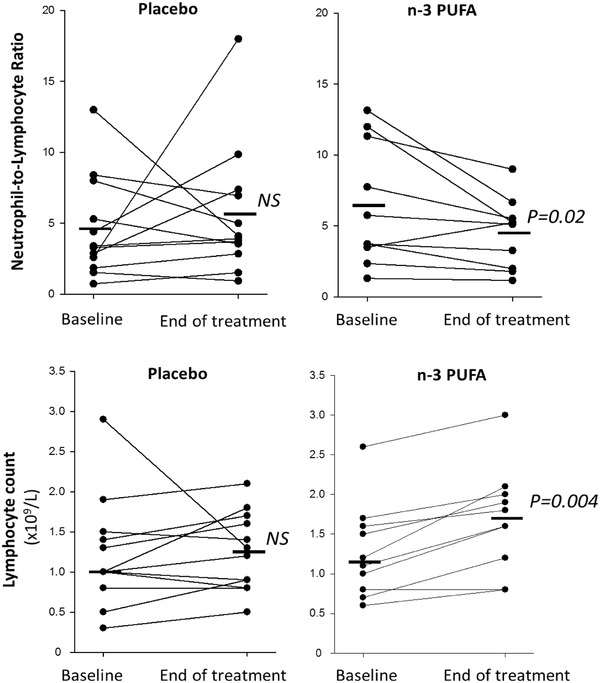
Neutrophil‐to‐lymphocyte ratio (upper panels) and lymphocyte counts (lower panels) at baseline and after treatment (End) with intravenous infusion (2 mL/kg) of either placebo (NaCl; left panels, n = 12) or n‐3 PUFA emulsion containing 10 g of fish oil per 100 mL (right panels, n = 10). Horizontal lines represent the median at each time point. Statistical analyses were performed with a Wilcoxon Signed Rank Test for repeated measures. NS, non‐significant. Statistical significance was set at *P* = 0.05

*n*‐3 PUFA treatment significantly increased plasma EPA‐ and DHA (Figure 2A) and their plasma metabolome, predominantly manifested for EPA‐derived metabolites (Supplementary Table 2). Arachidonic acid (AA; Figure 2A), its metabolome (Supplementary Table 3), and octadecanoid pathways from linoleic acid (LA), α‐LA, and γ‐LA (Supplementary Table 4) exhibted less alterations. Based on all PUFA metabolites, an orthogonal partial least squares discriminant analysis (OPLS‐DA) model evidenced a separation of the *n*‐3 PUFA and placebo groups with a statistic fit (R2 = 0.75) higher than its approximation (Q2 = 0.38) to a testing dataset (Figure 2B). Among the identified critical PUFA metabolites (Figure 2C), 18‐HEPE was significantly increased by n‐3 PUFA treatment (Figure 2D). 18‐HEPE is the precursor for short‐ lived, locally produced, specialized proresolving mediators (SPM), which raises a first suggestion of activated SPM‐induced resolution of inflammation by n‐3‐PUFA‐treatment of COVID‐19.

**FIGURE 2:**
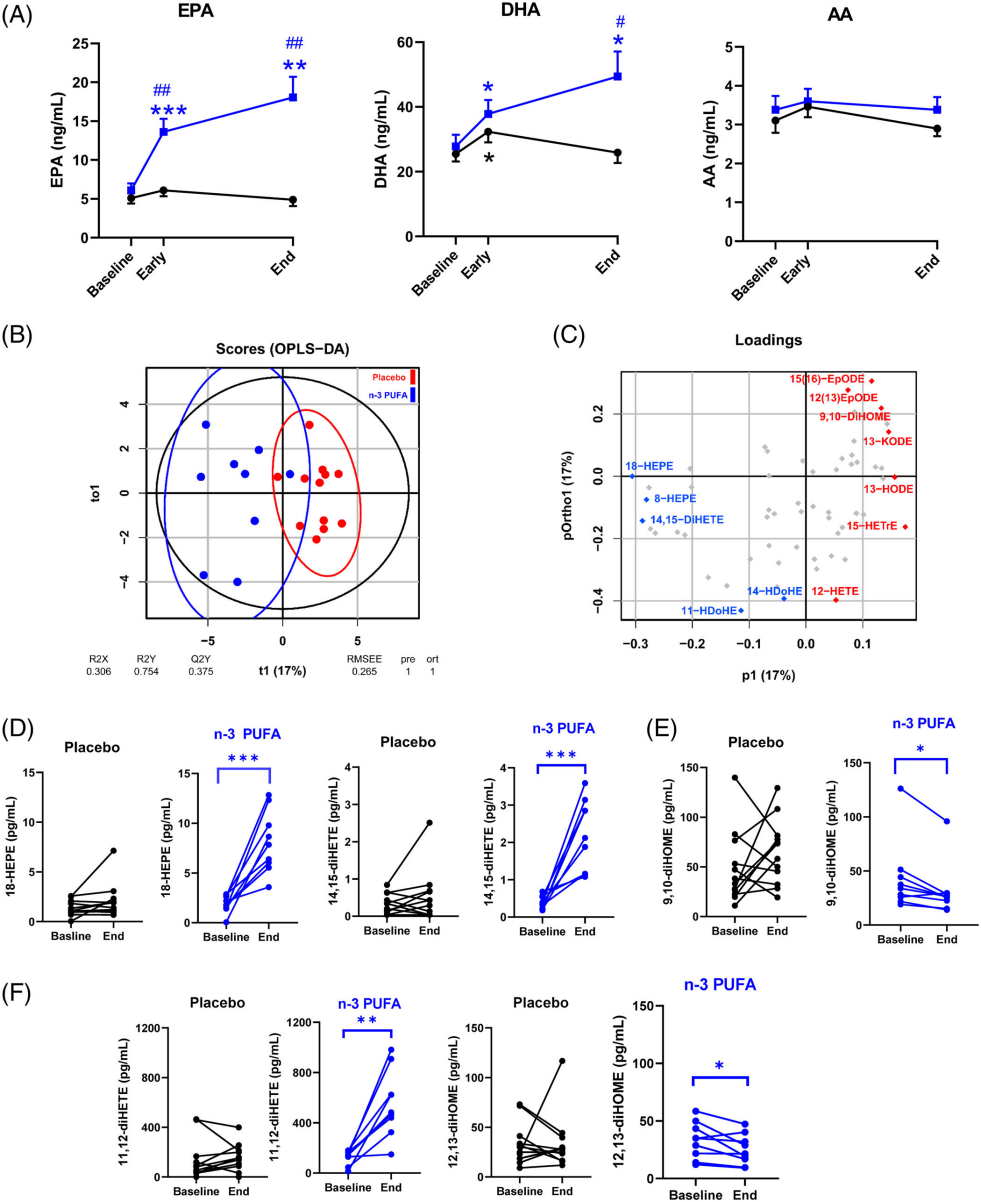
(A) Mass spectrometry analysis of circulating free fatty acids, eicosapentaenoic acid (EPA), docosahexanoic acid (DHA), and arachidonic acid (AA) in plasma from patients at baseline, 48 h (Early) and after treatment (End) following intravenous infusion (2 mL/kg) of either placebo (NaCl; black symbols, n = 12) or n‐3 PUFA emulsion containing 10 g of fish oil per 100 mL (blue symbols, n = 10) for 5 days. Results are expressed as mean ± SEM. Statistical analyses were performed with 2‐way ANOVA for repeated measures and post hoc testing. (B) Orthogonal partial least squares discriminant analysis (OPLS‐DA) separated the n‐3 PUFA (blue symbols) and placebo (red symbols). (C) The loading plot identified the EPA metabolites 18‐HEPE (left), 14,15‐diHETE (middle), and the LA metabolite 9,10‐diHOME (right) among the critical molecules explaining the differences in plasma lipid mediator profile caused by n‐3 PUFA intravenous infusion (blue = higher and red = lower in the n‐3 PUFA group), which were (D) significantly altered in the n‐PUFA (blue symbols; n = 9) but not placebo (black symbols; n = 12) groups between baseline and End. (E) Change in the LA‐derived leukotoxin diol 9,10‐diHOME (F) Change in the EPA and LA diols, 11,12‐diHETE (left) and 12,13‐diHOME (right). Results are presented as individual values and statistical analysis was performed by a paired Student's t test. Statistical significance was set at *P* = 0.05. *P < 0.05; **P < 0.01; ***P < 0.001 compared to baseline and ^#^ *P* < 0.05; ^##^ *P* < 0.01 between placebo and n‐3 PUFA treatment

In addition, the EPA‐derived diols 11,12‐ and 14,15‐diHETE (Figure 2D) increased, while the corresponding LA‐derived leukotoxin diols 9,10‐diHOME (Figure 2E) and isoleukotoxin diol 12,13‐diHOME (Figure 2F) decreased after n‐3 PUFA treatment. Double PUFA bond epoxidation followed by conversion to vicinal leukotoxin diols by the enzyme soluble epoxide hydrolase (sEH) are key effectors in adult respiratory distress syndrome (ARDS) and increased in COVID‐ 19 with severe pulmonary involvement.^5^ Decreased leukotoxin diols by *n*‐3 PUFA support the improved NLR (Figure 1) and the emerging notion of sEH as a COVID‐19 therapeutic target.^5^

Leukocyte–platelet aggregates, which aggravate ARDS^6^ and thrombotic complications of COVID‐19^7^, decreased by *n*‐3 PUFA (Figure 3A), driven by reduced neutrophil‐platelet aggregates (Figure 3B). Peripheral monocytic blood cells (PMBCs) isolated during and after n‐3 PUFA treatment released significantly lower levels of the platelet activators PDGF and RANTES (Figure 3C), indicating decreased immunothrombosis in COVID‐19 by n‐3 PUFA.

**FIGURE 3:**
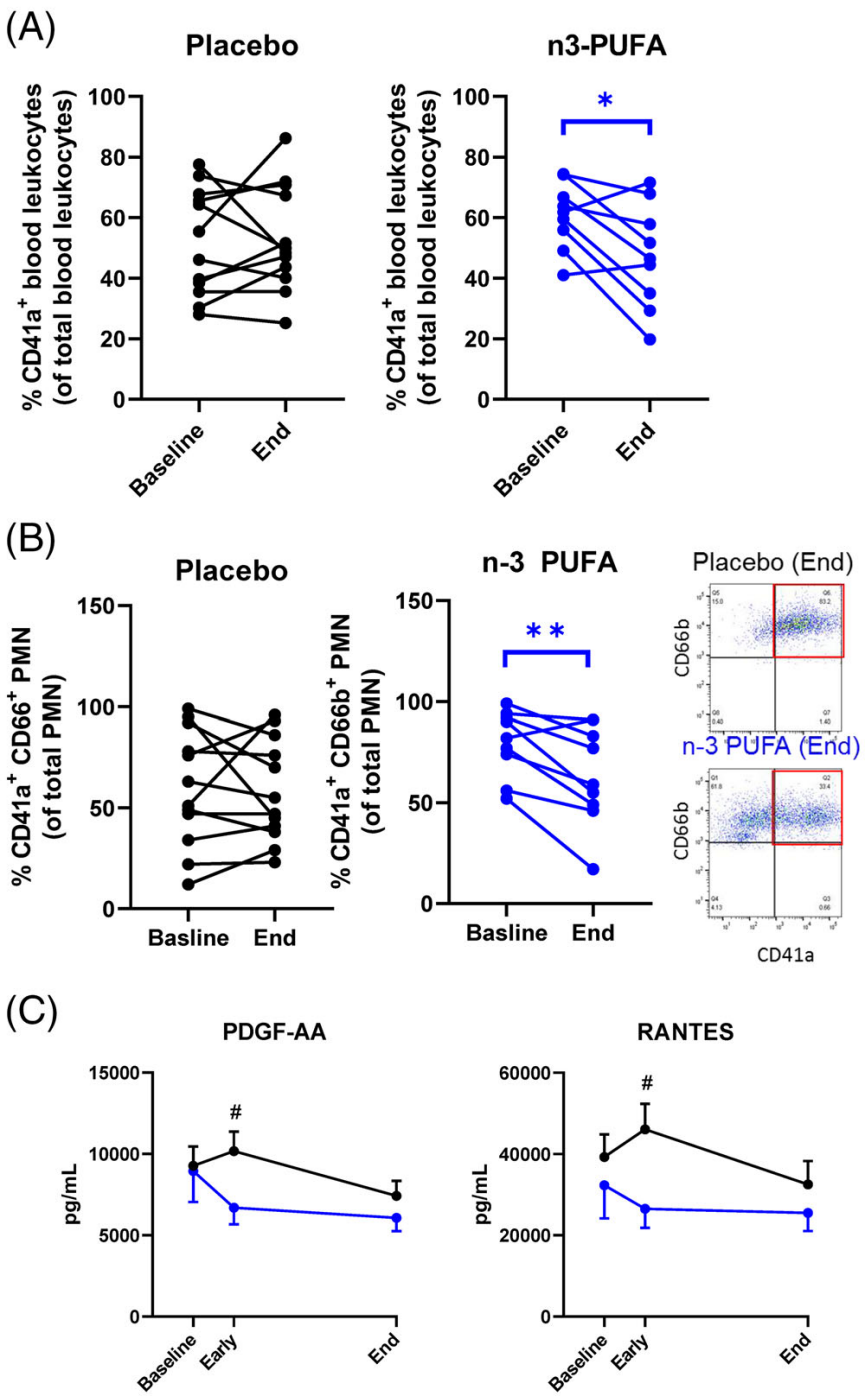
(A) Platelet‐leukocyte aggregates and (B) Platelet‐neutrophil aggregates at baseline and after treatment (End) with intravenous infusion (2 mL/kg) of either placebo (NaCl; black symbol, n = 12) or n‐3 PUFA emulsion containing 10 g of fish oil per 100 mL (blue symbol, *n* = 9). Results are presented as individual values and statistical analysis was performed by a paired Student's *t* test. Inset shows representative flow cytometry dot blots showing CD41^+^ CD66b^+^ platelet‐neutrophil aggregates in blood from Placebo (top) and n‐3 PUFA (bottom) treated patients. (C) The release of PBMC‐derived platelet activators, platelet derived growth factor (PDGF)‐AA and regulated on activation, normal T cell expressed and secreted, RANTES from LPS‐stimulated PBMC isolated from patients at baseline, at 48 h (Early), and after treatment (End) with intravenous infusion (2 mL/kg) of either placebo (black symbols NaCl; *n* = 12) or n‐3 PUFA emulsion containing 10 g of fish oil per 100 mL (blue symbols, *n* = 10). Results are expressed as mean ± SEM. Statistical analyses were performed with 2‐way ANOVA for repeated measures and post hoc testing. Statistical significance was set at *P* = 0.05. *P < 0.05; **P < 0.01 compared to baseline and ^#^ *P* < 0.05 between placebo and n‐3 PUFA treatment

The enhanced inflammation and more severe disease in older patients with comorbidities are linked to suppressed type 1 interferons (IFN‐1) α and β,^8^ which exhibited low release from PBMCs (Supplementary Fig 3). IFN‐1‐induced MX1 expression was retained in IFN‐responsive WISH cells incubated with plasma from *n*‐3 PUFA‐treated patients but time‐ dependently decreased in cells treated with placebo plasma (Figure 4A, left panel). Exogenous *n*‐3 PUFA stimulation did not alter the response (Figure 4A, middle panel), supporting systemic effects of *n*‐3 PUFA on IFN‐1 responses. The retained IFN‐1 response obtained by n‐3 PUFA administration for cortisone‐treated patients (Figure 4A, right panel) support prevention of cortisone‐induced immunosuppression^9^ by n‐3 PUFA treatment of COVID‐19.

**FIGURE 4:**
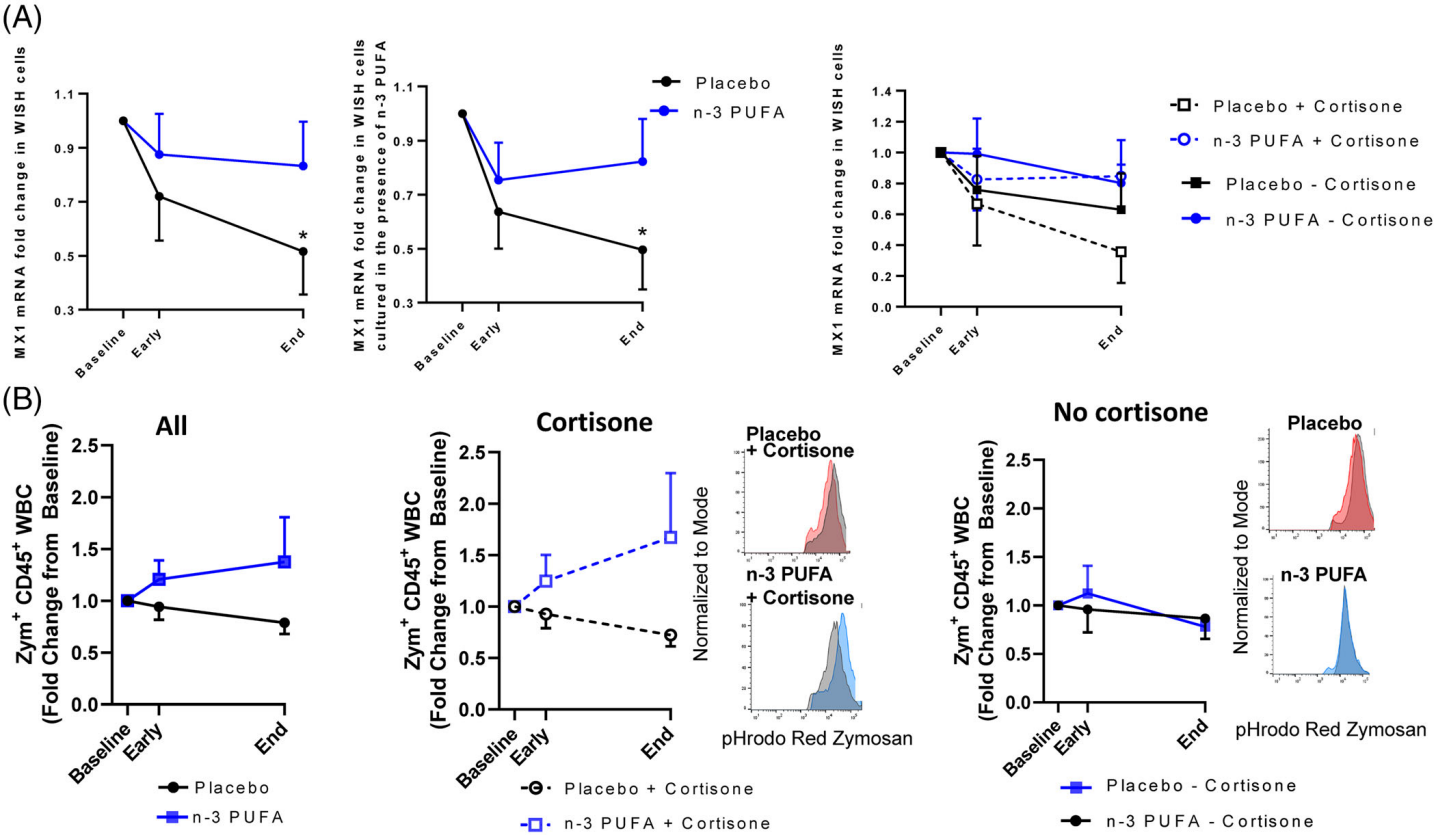
(A) RT‐qPCR analysis of MX1 mRNA expression in WISH cells stimulated with patient serum in the absence (right panel) or presence (middle panel) of exogenous n‐3 PUFA (placebo n = 12; n‐3 PUFA n = 10). Subgroups with (dotted line; placebo n = 6, n‐3 PUFA n = 7) and without (solid lines; placebo n = 6, n‐3 PUFA n = 3) concomitant cortisone treatment are shown in right panel. (B) Fold change in phagocytosis of pHrodo‐red labeled zymosan by human peripheral blood leukocytes (CD45^+^) after incubation for 45 min at 37°C (placebo n = 11; n‐3 PUFA n = 9). Subgroups with (middle panel; placebo n = 6, n‐3 PUFA n = 6) or without (right panel; placebo n = 5 and n‐3 PUFA n = 3) concomitant cortisone treatment Representative histograms from each group and subgroup at End are shown as inset. Plasma and whole blood were derived from patients at baseline, at 48 h (Early) and after treatment (End) with intravenous infusion (2 mL/kg) of either placebo (black symbols NaCl) or n‐3 PUFA emulsion containing 10 g of fish oil per 100 mL (blue symbols)

A decreased phagocyte function in COVID‐19 was restored in vitro after exogenous administration of *n*‐3 PUFA‐derived SPMs.^10^ The present study extends support for *n*‐3 PUFA as a promoter of the resolution of COVID‐19‐induced inflammation by a trend towards enhanced phagocytosis in leukocytes derived from n‐3 PUFA‐compared with placebo‐treated patients (Figure 4B, left panel). A more prominent phagocytosis increase by a combined cortisone and n‐3 PUFA treatment (Figure 4B; middle panel) compared to no concomitant cortisone (Figure 4B, right panel), further supports beneficial synergetic effects of cortisone and n‐3 PUFA treatment of COVID‐19 for retaining crucial immune functions and counteract cortisone‐induced immunosuppression.

There were 2 in‐hospital deaths in each group after randomization (*n* = 23; Figure S1). A descriptive analysis of length of hospital stay for the *n* = 22 subjects included in the analyses is shown in Figure S4. Serum triglyceride levels were not significantly increased, and no serious adverse events were encountered, supporting that i.v. *n*‐3 PUFA administration was safe and feasible during hospitalization for COVID‐19.

In summary, the primary outcome indicated a beneficial cellular immune response by i.v. *n*‐3 PUFA treatment of COVID‐19, along with increased proresolving mediator precursor levels and decreased leukotoxin‐diols. The exploratory cellular experiments showed decreased immunothrombosis, enhanced phagocytosis and retained IFN‐1 signaling by *n*‐3 PUFA‐treatment. The main limitation is the low number of participants and larger studies are needed to determine if the results translate into better clinical outcomes in COVID‐19. In conclusion, this proof‐of‐concept trial points to possible additive beneficial effects of *n*‐3 PUFA treatment in COVID‐19 on top of current treatment recommendations, in particular for vulnerable older COVID‐19 patients with multiple comorbidities, which tolerated and responded to *n*‐3 PUFA treatment.

## Supporting information

SUPPORTING MATERIAL
