# Supplementary material for: Immunomodulation by intravenous omega‐3 fatty acid treatment in older subjects hospitalized for COVID‐19: A single‐blind randomized controlled trial
Source: Clin Transl Med. 2022 Sep 19;12(9):e895. doi: 10.1002/ctm2.895 (PMC9484265; doi:10.1002/ctm2.895)
Supplement: Supplementary file 1 — SUPPORTING MATERIAL [file CTM2-12-0-s001.pdf]

Supplementary material

Immunomodulation by intravenous omega-3 fatty acid treatment in older subjects hospitalized for COVID-19: a single-blind randomized controlled trial

Hildur Arnardottir, *PhD*[1,*], Sven-Christian Pawelzik, *PhD* [1,*], Philip Sarajlic, *MD*[1], Alessandro Quaranta, *PhD*[2], Johan Kolmert, *PhD*[2,3], Dorota Religa, *MD PhD* [4], Craig E. Wheelock *PhD*[2,5], Magnus Bäck, *MD PhD* [1]

*Shared first author

[1]Department of Medicine, Karolinska Institutet, Theme Heart, Vessels, and Neuro, Karolinska University Hospital, Stockholm, Sweden
[2]Division of Physiological Chemistry 2, Department of Medical Biochemistry and Biophysics, Karolinska Institute, Stockholm, Sweden.
[3]The Institute of Environmental Medicine, Karolinska Institutet, Stockholm, Sweden.
[4]Department of Neurobiology, Karolinska Institutet and Theme Ageing, Karolinska University Hospital, Stockholm, Sweden
[5]Department of Respiratory Medicine and Allergy, Karolinska University Hospital, Stockholm, Sweden.

Registration in Trial Registries:
This trial "Resolving Inflammatory Storm in COVID-19 Patients by Omega-3 Polyunsaturated Fatty Acids - A single-blind, randomized, placebo-controlled feasibility study" (COVID-Omega-F) is registered in the European Union Drug Regulating Authorities Clinical Trials (EudraCT) database with number 2020-002293-28 and at Clinical Trials.gov with number NCT04647604.

Sources of Funding
The study received a research grant from King Gustaf V and Queen Victoria Freemason Foundation. The investigators were supported by the Swedish Research Council (Grant number 2019-01486) and the Swedish Heart and Lung Foundation (grant numbers 20180571, 20190625; 20190196; 20200693; 20210519). The sources of funding had no access to the study data and no role in the design, implementation, or reporting.

Conflicts of interest
None declared

Supplementary Methods

Patients

The trial "Resolving inflammatory storm in COVID-19 patients by Omega-3 Polyunsaturated fatty acids" (COVID-Omega-F) was approved by Swedish Ethical Review Authority on May 25, 2020 (Dnr 2020-02592) and by the Medical Product Agency on May 29, 2020 (Dnr 5.1-2020-42861). An amendment was approved to increase inclusion to achieve comparable groups completing the full study protocol according to the initial sample size calculations (Swedish Ethical Review Authority approval November 25, 2020; Dnr 2020-06137, and Medical Product Agency approval on November 30, 2020; Dnr 5.1-2020-96391). Inclusion criteria were COVID-19 diagnosis and clinical status requiring hospitalization. All subjects were included between from June to December 2020. After signed informed consent, participants were randomized 1:1 to a once daily i.v. infusion (2 mL/kg) of either placebo (0.9% NaCl) or n-3 PUFA emulsion (Omegaven® bought from ApoEX, Stockholm, Sweden) containing 10 g of fish oil per 100 mL, of which 1.25-2.82 g DHA and 1.44-3.09 g EPA for 5 days. Randomization was performed using sequentially numbered sealed envelopes in random permuted blocks of n=2-3. Participants were blinded to the intervention. The sample size was determined to at least 10 patients in each group based on previous studies of i.v. PUFA emulsions on infectious inflammation (1). The primary endpoint measures were changes in inflammatory biomarkers (time frame: after either 5 days treatment or at study end, whichever came first) defined as *(I)* white blood cell counts, *(II)* C-reactive protein (CRP), *(III)* lipidomic profiling, and *(IV)* cytokines.

Blood and urine sample collection

Laboratory measures of routine biochemistry, blood cell counts, and CRP were performed by the Karolinska University Laboratory in accordance with ISO15189. Blood and urine samples for biomarker and lipid metabolite analyses were collected at the study start before administration of the first dose of treatment (baseline), at 24-48h after the first administered dose (early), and within 24h of the last administered dose (end). Plasma was used for measurements of markers of inflammation using the multiplex Ella™ system (ProteinSimple, Biotechne, San Jose, CA, USA).

Lipid metabolite analysis

Lipid mediator quantification was performed as previously described (2) with minor modifications to the sample preparation to account for the solvent composition. One mL MeOH was added to 250 µL plasma and samples were vortexed and centrifuged. The supernatant was transferred to glass tubes and evaporated under gentle N_2 stream to remove the MeOH before reconstitution in 1 mL of extraction solution (0.2 M Na_2HPO_4 / 0.1 M citric acid, pH 5.6) followed by solid phase extraction as previously reported (2). Twelve additional compounds were included in the current study that were not reported in the previous methods description, including epoxides and vicinal diols from AA and DHA (Supplementary Table 5). Non-commercially epoxide and vicinal diol analytical standards were kindly provided by Bruce Hammock at the University of California Davis (CA, USA) and synthesized by Dr Johanna Revol-Cavalier at Karolinska Institutet.

Blood cell isolation

Whole blood was collected into an 8 mL sodium heparinized CPT vacutainer and processed within 2 h of collection. Peripheral blood mononuclear cells (PBMC) were separated from erythrocytes and neutrophils following centrifugation at 1800 x g for 15 min at room temperature. Erythrocytes were isolated by 3% dextran sedimentation for 20 minutes at room temperature and then washed 3 times with $PBS^{-/-}$ (followed each time by centrifugation at 1000 x g, 10 min, 4°C).

PBMC stimulations for mediator release measurements

PBMC were collected as described above, washed with $PBS^{-/-}$ followed by centrifugation for 10 min at 250 x g, 4°C, and then resuspended in RPMI media containing 1% heat-inactivated (56°C for 30 min) autologous serum at a concentration of 2.5×10^6 cells/mL. 0.5×10^6 cells/well were seeded into a round bottom 96-well plate and incubated with or without 1 µg/mL LPS (serotype O111:B4, Sigma-Aldrich, Merck KGaA, St. Louis, MO, USA) for 6 h. Following centrifugation for 5 min at 300 x g, 4°C, the supernatants were collected and stored at -80°C.

Luminex

Supernatants from stimulated and unstimulated PBMC were used for measurements of markers of inflammation using Human XL Cytokine Luminex Performance Assay multiplex kit (RnD Systems, Minneapollis, MO, USA).

Flow cytometric analysis of stimulated whole blood

Fresh whole blood was obtained from sodium heparinized CPT vacutainers prior to centrifugation for whole blood functional assays. For phagocytosis assessment, 30 µl whole blood was incubated with opsonized pH sensitive zymosan particles (pHrodo™ Red, Invitrogen, Thermo Fisher Scientific, Waltham, MA, USA) for 60 min at 37°C or 4°C (negative control). Samples were then incubated with APC-conjugated anti-human CD66b antibody, FITC-conjugated anti-human CD14 antibody, and eFluor450-conjugated anti-human CD45 antibody (all from Thermo Fisher Scientific, Waltham, MA, USA) for 15 min on ice. For platelet-leukocyte aggregation assessment, 45 µl whole blood were incubated with 10 µM ADP (Sigma-Aldrich, Merck KGaA, St. Louis, MO, USA) for 10 minutes at 37°C. Samples were then incubated with APC-conjugated anti-human CD66b antibody, PE-conjugated anti-human CD41a antibody, FITC-conjugated anti-human CD14 antibody, and eFluor450-conjugated anti-human CD16 antibody (all from Thermo Fisher Scientific, Waltham, MA, USA) for 15 min at RT. All samples were fixed and lysed using 1-step Fix/Lyse solution (eBioscience, Thermo Fisher Scientific, Waltham, MA, USA) and washed 2 time with Flow Cytometry Staining Buffer (eBioscience, from Thermo Fisher Scientific, Waltham, MA, USA). Cells were analyzed with BD Fortessa flow cytometer (BD Biosciences) and Flow Jo software version v10.7.0.

Measurement of biological interferon activity

An interferon (IFN) sensitive human amniotic epithelial cell line (WISH, ATCC® CCL-25™) was used to assess the biological activity of type I (IFN-α and IFN-β) and type II (IFN-γ) IFN, respectively, in patient serum as previously described (3). The commercially available WISH cell line is contaminated with HeLa cells (4). In brief, 1.25×10^5 cells/well in a total volume of 200 µl MEM

supplemented with 5% heat-inactivated FBS, 2 mM L-glutamine, 1 mM Na-pyruvate, 10 mM HEPES, 50 IU/ml penicillin, and 50 µg/ml streptomycin (all from Thermo Fisher Scientific, Waltham, MA, USA), were seeded into a 96-well plate and cultured over night at 37°C in a humified 5% CO_2 atmosphere. At the day of the experiment, the medium was aspirated and replaced with 200 µl MEM supplemented with 50% patient sera. Control cells were incubated with serum-free medium containing 0-200 IU/ml recombinant human IFN-α, IFN-β, and IFN-γ, respectively (RnD Systems, Minneapolis, MO, USA). After incubation for 6 h at 37°C in a humified 5% CO_2 atmosphere, the media were aspirated, the cells were lyzed in 350 µl RLT buffer (Qiagen, Hilden, Germany) supplemented with 10 µl/ml β -mercaptoethanol and frozen at -80°C until further analysis. Total RNA was extracted using the RNeasy Mini Kit (Qiagen, Hilden, Germany) following the manufacturer's instruction and subsequently reverse transcribed into cDNA using the High-Capacity RNA-to-cDNA Kit (Thermo Fisher Scientific, Waltham, MA, USA). Expression levels of the type I IFN-induced response gene MX1 and the type II IFN-induced response gene CXCL9 were analyzed by quantitative real time PCR (qPCR) on a QuantStudio 7 Flex Real-Time PCR System using specific TaqMan Gene expression assays (MX1, Hs00895608_m1; CXCL9, Hs00171065_m1, all from Thermo Fisher Scientific, Waltham, MA, USA) and quantified by comparison to the expression levels in the respective standard curves obtained from cells that were incubated with recombinant IFN protein. The procedure was replicated with WISH cells that were cultured for three consecutive passages prior to the experiment in MEM supplemented with 0.01% n-3 PUFA emulsion (v/v) to enrich n-3 PUFA in the cell membrane.

Statistical analysis

Outcome measures are expressed as means with standard errors. Key results are presented as separate line plots for the placebo and n-3 PUFA groups showing changes within subjects between baseline and the endpoint. Paired t-tests were used to determine significance in changes over time. To determine the effect of time and the n-3 PUFA intervention, a mixed model analysis of variance was implemented in which time was defined as a within-subjects effect and the intervention as a between-subjects effect. Moreover, the interaction between these effects was measured. Due to the limited statistical power of the study, descriptive outcomes were not

tested for significance as defined in the prespecified analysis plan in the study protocol, which is provided as an Appendix online. To gain insight into the key lipid metabolites separating the placebo and n-3 PUFA intervention groups, we implemented an orthogonal partial least squares discriminant analysis (OPLS-DA) model to analyze the variation correlated to the intervention. The residual variation is projected upon the components that are orthogonal to the main component that is predictive of the target of interest. The model generated variable statistic (R2) and approximation (Q2) importance scores to identify what metabolites accounted for the greatest differences in the placebo and n-3 PUFA groups. The length of hospital stay was calculated as the time to discharged alive up to 30 days of hospitalization, after assigning time ∞ to in-hospital death cases (5), and was expressed as the proportion of subjects included in each group (n=12 placebo and n=10 n-3 PUFA; Supplementary Fig 1). All significance tests were two-sided and findings with $P<0.05$ were regarded as statistically significant. Statistical analyses were performed in R version 4.1.1 (R Core Team 2021). R: A language and environment for statistical computing. R Foundation for Statistical Computing, Vienna, Austria. URL https://www.R-project.org/), Bioconductor version 3.13 (6), GraphPad (version 8.4.3, Sand Diego, California, USA), and SigmaPlot for Windows Version 14.5.

References

1. Mayer K, Gokorsch S, Fegbeutel C, Hattar K, Rosseau S, Walmrath D, Seeger W, Grimminger F. Parenteral nutrition with fish oil modulates cytokine response in patients with sepsis. *Am J Respir Crit Care Med* 2003; 167: 1321-1328.
2. Kolmert J, Fauland A, Fuchs D, Safholm J, Gomez C, Adner M, Dahlen SE, Wheelock CE. Lipid Mediator Quantification in Isolated Human and Guinea Pig Airways: An Expanded Approach for Respiratory Research. *Anal Chem* 2018; 90: 10239-10248.
3. Hua J, Kirou K, Lee C, Crow MK. Functional assay of type I interferon in systemic lupus erythematosus plasma and association with anti-RNA binding protein autoantibodies. *Arthritis Rheum* 2006; 54: 1906-1916.
4. Ross MG, Wang S. Wishing the WISH cells were pure. *Am J Obstet Gynecol* 2003; 189: 1807-1808; author reply 1808.
5. Brock GN, Barnes C, Ramirez JA, Myers J. How to handle mortality when investigating length of hospital stay and time to clinical stability. *BMC Med Res Methodol* 2011; 11: 144.
6. Huber W, Carey VJ, Gentleman R, Anders S, Carlson M, Carvalho BS, Bravo HC, Davis S, Gatto L, Girke T, Gottardo R, Hahne F, Hansen KD, Irizarry RA, Lawrence M, Love MI, MacDonald J, Obenchain V, Oles AK, Pages H, Reyes A, Shannon P, Smyth GK, Tenenbaum D, Waldron L, Morgan M. Orchestrating high-throughput genomic analysis with Bioconductor. *Nat Methods* 2015; 12: 115-121.

Supplementary Table 1: Baseline characteristics

	All	*Placebo*	*n-3 PUFA*	*P*
n	*22*	*12*	*10*	
Age - years	*81.1±6.1*	*81.5±5.6*	*80.7±7.0*	*0.77*
Female sex – no. (%)	*12 (55%)*	*7 (58%)*	*5 (50%)*	*0.70*
Body Mass Index - kg/m^2	*25.6±3.2*	*25.0±5.6*	*26.3±4.4*	*0.46*
Current smoker – no. (%)	*2 (9%)*	*0 (0%)*	*2 (20%)*	*0.20*
Days since symptom start	*7.1±3.3*	*7.2±3.5*	*7.0±3.1*	*0.87*
Days since COVID-19 diagnosis	*3.5±1.7*	*3.5±2.0*	*3.7±1.3*	*0.83*
***Medical history** – no. (%)*				
Cardiovascular disease	*14 (64%)*	*9 (75%)*	*5 (50%)*	*0.38*
Hypertension	*15 (64%)*	*9 (75%)*	*6 (60%)*	*0.65*
COPD/Asthma	*3 (13%)*	*1 (8.3%)*	*2 (20%)*	*0.57*
Cancer	*4 (18%)*	*2 (17%)*	*2 (20%)*	*1.0*
Diabetes	*9 (41%)*	*3 (24%)*	*6 (60%)*	*0.19*
CKD	*5 (23%)*	*2 (17%)*	*3 (30%)*	*0.62*
Rheumatological disease	*4 (18%)*	*2 (17%)*	*2 (20%)*	*1.0*
***Concomitant medication** – no. (%)*				
Acetyl salicylic acid	*8 (36%)*	*6 (50%)*	*2 (20%)*	*0.20*
Clopidogrel	*3 (14%)*	*2 (17%)*	*1 (10%)*	*1.0*
Agents acting on the RAS	*9 (41%)*	*5 (42%)*	*4 (40%)*	*1.0*
Beta blockers	*11 (50%)*	*5 (42%)*	*6 (60%)*	*0.67*
Calcium channel blockers	*3 (14%)*	*2 (17%)*	*1 (10%)*	*1.0*
Diuretics	*5 (23%)*	*3 (26%)*	*2 (20%)*	*1.0*
Nitrates	*2 (9%)*	*0 (0%)*	*2 (20%)*	*0.20*
Statins	*10 (45%)*	*5 (42%)*	*5 (50%)*	*1.0*
DOAC	*5 (23%)*	*1 (8%)*	*4 (40%)*	*0.14*
Insulin	*4 (18%)*	*2 (17%)*	*2 (20%)*	*1.0*
Oral anti-diabetics	*5 (23%)*	*1 (8%)*	*4 (40%)*	*0.14*
ICS	*3 (14%)*	*1 (8%)*	*2 (20%)*	*0.57*
Blood Cell Counts				
Leukocytes ($x10^9/L$)	*6.7 (4.7-9.6)*	*5.6 (3.7-9.2)*	*8.1 (6.1-11)*	*0.09*
Monocytes ($x10^9/L$)	*0.6 (0.3-0.8)*	*0.45 (0.30-0.78)*	*0.65 (0.50-0.85)*	*0.26*
Neutrophils ($x10^9/L$)	*5.0 (3.2-6.7)*	*4.0 (2.3-6.3)*	*5.6 (4.1-9.2)*	*0.06*
Lymphocytes ($x10^9/L$)	*1.1 (0.8-1.5)*	*1.0 (0.85-1.48)*	*1.15 (0.78-1.62)*	*0.72*
NLR	*3.7 (2.5-8.1)*	*3.3 (2.1-7.3)*	*4.7 (3.2-11.5)*	*0.28*
Erythrocytes ($x10^{12}/L$)	*4.1 (3.4-4.5)*	*4.2 (3.4-4.7)*	*4.0 (3.4-4.3)*	*0.36*

Supplementary Table 1: Baseline characteristics

EVF	*0.36 (0.32-0.40)*		*0.35 (0.30-0.40)*	*0.36 (0.34-0.39)*		*0.70*
Platelets (x10^9/L)	*226 (168-292)*		*217 (162-306)*	*240 (190-289)*		*0.90*
Coagulation, Thrombosis						
Fibrin-D-dimer (mg/L)	*0.71 (0.57-1.78)*		*0.61 (0.51-0.92)*	*1.1 (0.67-1.9)*		*0.08*
Fibrinogen (g/L)	*5 (3.9-5.6)*		*5.0 (4.1-5.3)*	*4.8 (3.5-6.2)*		*0.92*
APTT (s)	*26 (23-29)*		*24 (23-26)*	*25 (22-34)*		*0.65*
INR	*1.1 (1.0-1.2)*		*1.1 (1.0-1.1)*	*1.1 (1.1-1.4)*		*0.33*
Organ Damage						
ALAT (μcat/L)	*0.34 (0.27-0.76)*		*0.33 (0.23-0.68)*	*0.36 (0.28-1.31)*		*0.45*
ASAT (μcat/L)	*0.43 (0.36-0.83)*		*0.45 (0.37-0.83)*	*0.43 (0.28-0.89)*		*0.95*
LD (μcat/L)	*5.0 (3.8-6.2)*		*5.0 (4.0-6.9)*	*4.8 (3.6-5.8)*		*0.55*
Bilirubin (μmol/L)	*7 (6-12)*		*6 (5-10)*	*9.5 (6-16)*		*0.09*
Creatinine (μmol/L)	*72 (59-106)*		*80 (59-95)*	*65 (55-114)*		*0.90*
eGFR (mL/min/1.73 m^2)	*59 (45-77)*		*59 (47-74)*	*61 (45-81)*		*0.87*
Troponin T (ng/L)	*19 (13-39)*		*15 (13-47)*	*24 (13-33)*		*0.92*
Chemistry						
Hb (g/L)	*116 (102-129)*		*111 (96-132)*	*120 (106-129)*		*0.60*
Ferritin (μmol/L)	*614 (223-810)*		*674 (213-799)*	*498 (232-1578)*		*0.88*
Na (mmol/L)	*138 (136-140)*		*139.5 (136- 140)*	*137 (134-140)*		*0.50*
K (μmol/L)	*3.9 (3.7-4.3)*		*3.8 (3.7-4.1)*	*4.2 (3.8-4.5)*		*0.20*
Ca (μmol/L)	*2.21 (2.12-2.29)*		*2.21 (2.12-2.29)*	*2.21 (2.12-2.29)*		*0.80*
Albumin (g/L)	*28 (26-32)*		*30 (27-33)*	*28 (24-30)*		*0.26*

Supplementary Table 1. Patient characteristics. Demographic data are expressed as either mean ± standard deviation or numbers and per cent. Laboratory measures are expressed as median (interquartile range). Statistical analyses of demographic data were performed by either a Student's t-test (continuous variables) or a Fisher Exact Test (categorical variables). Cardiovascular disease was defined as medical history of at least one of the following: coronary artery disease, cerebrovascular disease, peripheral artery disease, heart failure, atrial fibrillation, venous thromboembolism. All laboratory measures were carried out by Karolinska University Laboratory in accordance with ISO15189. Statistical analyses of laboratory data were performed using a Mann-Whitney Rank Sum Test. *Abbreviations: ALAT, alanine aminotransaminase; APTT, activated partial thromboplastin time; ASAT, aspartate aminotransferase; COPD, chronic obstructive pulmonary disease; DOAC, direct oral anticoagulants, eGFR, estimated glomerular filtration rate; EVF, erythrocyte volume fraction, ICS, inhaled corticosteroids, LD, lactate dehydrogenase; NLR, neutrophil-to-lymphocyte ratio; RAS, renin-angiotensin system.*

Supplementary Table 2

	Baseline	Early	End		Baseline	Early	End		Interv	Progress	Interv:Progr
EPA Metabolites											
PGE₃	0.08 (0.06)	0.14 (0.08)	0.36 (0.16)		0.05 (0.03)	0.11 (0.06)	0.02 (0)		0.064	0.394	0.051
TXB₃	0.08 (0)	0.83 (0.5)	0.5 (0.42)		0.08 (0)	0.08 (0)	0.08 (0)		0.13	0.258	0.258
5-HEPE	3.96 (0.77)	6.28 (0.88)	7.9 (1.39)		3.3 (0.49)	3.38 (0.41)	3.08 (0.55)		0.002	0.181	0.058
8-HEPE	0.39 (0.06)	1.1 (0.15)	2.2 (0.32)		0.32 (0.08)	0.29 (0.06)	0.51 (0.2)		0.0000175	0.000639	0.014
9-HEPE	0.2 (0.07)	0.83 (0.27)	1.97 (0.34)		0.25 (0.08)	0.19 (0.07)	0.25 (0.13)		0.0000727	0.003	0.005
11-HEPE	0.26 (0.06)	0.7 (0.11)	1.44 (0.27)		0.2 (0.07)	0.2 (0.08)	0.26 (0.09)		0.0000927	0.002	0.005
12-HEPE	20.89 (4.53)	34.37 (6.03)	60.9 (18.62)		13.5 (2.71)	17.36 (4.3)	18.1 (4.59)		0.007	0.117	0.138
15-HEPE	5.05 (1.63)	7.16 (1.69)	7.17 (1.11)		1.72 (0.57)	2.96 (0.82)	3.1 (0.96)		0.015	0.87	0.892
18-HEPE	1.86 (0.29)	5.33 (0.59)	8.11 (1.04)		1.38 (0.22)	1.9 (0.17)	2.12 (0.49)		0.0000044	0.001	0.005
8(9)-EpETE	12.88 (12.86)	5.76 (5.74)	26.1 (26.08)		5.09 (5.07)	25.4 (18.9)	0.02 (0)		0.839	0.877	0.171
11(12)-EpETE	88.07 (45.09)	208.73 (79.04)	329.04 (77.2)		143. (56.4)	83.1 (63.5)	94.8 (40.5)		0.033	0.175	0.26
14(15)-EpETE	0.05 (0.02)	0.26 (0.13)	0.27 (0.08)		0.12 (0.05)	0.04 (0)	0.04 (0)		0.014	0.937	0.937
17(18)-EpETE	0.22 (0.15)	1.35 (0.39)	1.7 (0.44)		0.2 (0.13)	0.13 (0.11)	0.32 (0.21)		0.003	0.073	0.584
5,6-DiHETE	0.16 (0.06)	0.08 (0)	0.09 (0.01)		0.08 (0)	0.08 (0)	0.08 (0)		0.11	0.258	0.258
11,12-DiHETE	125.46 (19.19)	439.42 (80.77)	555.99 (88.35)		129 (47.1)	102 (34.8)	145 (33.1)		0.0000965	0.023	0.267
14,15-DiHETE	5.05 (0.42)	9.54 (1.54)	13.1 (1.76)		4.73 (0.88)	3.9 (0.78)	4.16 (0.77)		0.0000733	0.037	0.069
17,18-DiHETE	39.95 (4.84)	84.24 (14.08)	116.99 (20.56)		40.0 (4.12)	41.3 (3.7)	41.3 (4.66)		0.000842	0.006	0.006
DHA Metabolites											
8-HDoHE	3.38 (0.53)	4.09 (0.76)	6.28 (1.03)		2.41 (0.23)	3.03 (0.3)	2.99 (0.33)		0.011	0.013	0.01
11-HDoHE	5.03 (0.82)	4.94 (0.89)	8.21 (1.97)		2.94 (0.63)	4.36 (0.97)	4.38 (0.93)		0.124	0.126	0.132
14-HDoHE	62.54 (11.02)	61.9 (14.97)	96.86 (28.46)		48.0 (7.94)	68.7 (17.0)	69.4 (16.4)		0.652	0.273	0.292
17-HDoHE	36.82 (6.91)	43.17 (5.51)	41.93 (4.14)		23.3 (3.29)	33.6 (4.82)	36.0 (5.7)		0.267	0.843	0.545
7(8)-EpDPA	99.62 (58.02)	161.95 (81.96)	152.14 (79.64)		85.6 (36.4)	39.6 (24.5)	79.5 (36.5)		0.19	0.635	0.436
10(11)-EpDPA	1604.71 (237.47)	2142.73 (373.51)	2201.72 (318.09)		1810 (294)	1817 (240)	1774 (312)		0.392	0.93	0.603
16(17)-EpDPA	74.56 (53.14)	133.47 (56.71)	191.08 (59.28)		30.5 (24.7)	58.9 (26.02)	30.8 (23.3)		0.035	0.564	0.104
19(20)-EpDPA	1.84 (0.67)	4.4 (1.55)	5.75 (1.57)		2.52 (0.54)	2.95 (0.5)	3.3 (0.75)		0.196	0.09	0.301
7,8-DiHDPA	114.17 (58.64)	144.49 (80.12)	354.16 (140.23)		65.4 (44.6)	99.3 (52.2)	132 (59.3)		0.134	0.15	0.285

Supplementary Table 2

10,11-DiHDPA	18.62 (18.6)	33.73 (27.84)	48.07 (25.37)		9.03 (9.01)	6.99 (5.82)	3.79 (2.08)		0.131	0.401	0.192
16,17-DiHDPA	18.61 (10.97)	78.29 (35.56)	96.12 (35.85)		76.5 (24.0)	72.4 (26.8)	54.7 (17.3)		0.529	0.998	0.286
19,20-DiHDPA	12.77 (2.07)	18.29 (2.57)	24.48 (4.81)		16.15 (1.6)	16.71 (1.6)	15.7 (1.16)		0.127	0.153	0.05
16,17-DiHDPA	18.61 (10.97)	78.29 (35.56)	96.12 (35.85)		76.5 (24.0)	72.4 (26.8)	54.7 (17.3)		0.529	0.998	0.286
10,11-DiHDPA	18.62 (18.6)	33.73 (27.84)	48.07 (25.37)		9.03 (9.01)	6.99 (5.82)	3.79 (2.08)		0.131	0.401	0.192

Supplementary Table 2. Lipid mediator metabolites (pg/mL) from the n-3 PUFA EPA and DHA in patient plasma. P-values were derived from ta mixed effects ANOVA containing intervention (Interv; n-3 PUFA vs. placebo) and progress (early and end samples) as a within-subjects effect and as a between subjects effect. Intervention:progress (Interv:Prog) represents the significance of the interaction between these two terms
Abbreviations: PG, Prostaglandin; TX, Abbreviations: PG, Prostaglandin; TX, thromboxane; EPE, Hydroxyeicosapentaenoic acid; EpETE, Epoxyeicosatetraenoic acid. DiHETE, Dihydroxyeicosatetraenoic acid; HDoHE, Hydroxydocosahexaenoic acid; HDoHE, Hydroxydocosahexaenoic

Supplementary Table 3: Plasma AA metabolites

	n-PUFA (n=9)			Placebo (n=12)			P-values		
	Baseline	Early	End	Baseline	Early	End	Interv	Progress	Interv:Progr
AA Metabolome									
20-HETE	7.35 (1.9)	10.13 (1.7)	9.48 (1.49)	10 (2.38)	11.55 (2.06)	10.6 (2.55)	0.641	0.582	0.91
19-HETE	7.24 (0.99)	8 (1.44)	7.54 (0.8)	7.05 (0.92)	7.92 (1)	6.58 (0.82)	0.688	0.207	0.534
15-HETE	11.93 (2.24)	10.43 (1.8)	7.9 (0.73)	7.22 (0.95)	8.81 (1.28)	9.4 (1.46)	0.971	0.243	0.068
12-HETE	352.2 (79.2)	225.4 (46.8)	309 (71.4)	257 (51.0)	329 (80.6)	313 (59.3)	0.525	0.511	0.332
11-HETE	3.23 (0.28)	3.1 (0.43)	3.42 (0.66)	2.84 (0.54)	3.33 (0.71)	3.36 (0.7)	0.923	0.445	0.522
9-HETE	1.04 (0.51)	1.01 (0.51)	1.99 (0.47)	0.89 (0.37)	1.58 (0.4)	1 (0.36)	0.704	0.487	0.012
8-HETE	4.76 (0.62)	4.6 (0.75)	4.87 (0.68)	3.61 (0.39)	4.29 (0.4)	4.13 (0.27)	0.437	0.865	0.494
5-HETE	33.28 (5.77)	35.73 (9.05)	23.96 (4.58)	18.5 (1.93)	22.68 (2.83)	19.2 (1.93)	0.119	0.072	0.314
15-KETE	1.2 (0.16)	1.06 (0.16)	0.89 (0.16)	0.78 (0.16)	0.81 (0.18)	0.84 (0.15)	0.5	0.465	0.284
12-KETE	1.23 (0.27)	0.8 (0.16)	0.65 (0.2)	0.97 (0.27)	1.36 (0.28)	0.92 (0.18)	0.146	0.08	0.367
5-KETE	1.16 (0.3)	1.4 (0.44)	1.14 (0.23)	1.11 (0.1)	0.79 (0.19)	1.1 (0.18)	0.326	0.903	0.133
PGE_2	3.73 (0.98)	3.39 (0.83)	5.95 (1.63)	2.92 (1.26)	4.38 (1.95)	4.95 (1.67)	0.997	0.063	0.224
13,14-dihydro-15-keto-PGE_2	0.14 (0.06)	0.19 (0.07)	0.12 (0.05)	0.11 (0.03)	0.15 (0.06)	0.14 (0.05)	0.854	0.425	0.486
8-isoPGE_2	0.3 (0.06)	0.5 (0.31)	0.21 (0.11)	0.18 (0.06)	0.36 (0.2)	0.19 (0.07)	0.734	0.1	0.668
PGD_2	1.01 (0.37)	0.81 (0.33)	2.36 (0.6)	1.01 (0.73)	1.56 (0.75)	1.9 (0.76)	0.879	0.003	0.041
$PGF_{2\alpha}$	7.06 (1.3)	6.07 (1.21)	10.65 (2.62)	4.63 (1.73)	6.76 (2.49)	7.14 (1.89)	0.626	0.061	0.109
PGB_2	0.37 (0.07)	0.42 (0.12)	0.53 (0.16)	0.36 (0.09)	0.31 (0.07)	0.39 (0.07)	0.384	0.073	0.762
12-HHTrE	14.0 (5.49)	18.8 (8.81)	21.5 (8.51)	20.0 (13.3)	22.0 (14.2)	25.7 (13.8)	0.831	0.558	0.917
TXB_2	12.3 (5.33)	18.81 (9.2)	19.27 (8.35)	11.6 (6.27)	15.79 (9.65)	23.4 (10.2)	0.966	0.483	0.534
11-keto-TXB_2	0.08 (0)	0.08 (0)	0.08 (0)	0.09 (0.01)	0.09 (0.02)	0.09 (0.01)	0.227	0.823	0.823
LTE_4	2.09 (0.94)	2.98 (2.32)	1.07 (0.33)	0.82 (0.17)	1.4 (0.54)	1.11 (0.51)	0.533	0.266	0.414
LTD_4	0.36 (0.13)	0.29 (0.19)	0.14 (0.08)	0.17 (0.07)	0.32 (0.14)	0.21 (0.11)	0.784	0.218	0.869
LTC_4	2.67 (0.75)	2.62 (0.74)	3.01 (0.68)	3.74 (0.47)	4.02 (0.79)	2.3 (0.73)	0.684	0.346	0.141
LTB_4	4.79 (1.34)	6.1 (3.52)	2.42 (0.78)	1.96 (0.31)	2.25 (0.59)	1.87 (0.44)	0.216	0.186	0.277
6-trans-LTB_4	0.47 (0.14)	0.43 (0.21)	0.14 (0.06)	0.11 (0.03)	0.13 (0.04)	0.1 (0.03)	0.13	0.099	0.18

Supplementary Table 3: Plasma AA metabolites

	n-PUFA (n=9)			Placebo (n=12)			P-values		
20-COOH-LTB_4	4.7 (1.92)	4.78 (2.35)	1.47 (0.58)	0.91 (0.39)	1.56 (0.59)	1.42 (0.56)	0.183	0.135	0.167
20-OH-LTB_4	1.33 (0.5)	1.03 (0.59)	0.37 (0.11)	0.61 (0.28)	0.57 (0.21)	0.35 (0.14)	0.478	0.103	0.406
5,15-DiHETE	0.09 (0.05)	0.1 (0.06)	0.02 (0)	0.05 (0.03)	0.07 (0.03)	0.11 (0.06)	0.587	0.613	0.143
14(15)-EpETrE	0.62 (0.25)	0.64 (0.29)	0.58 (0.24)	0.74 (0.2)	0.77 (0.21)	0.59 (0.22)	0.813	0.496	0.727
11(12)-EpETrE	0.31 (0.09)	0.36 (0.1)	0.26 (0.08)	0.33 (0.1)	0.25 (0.07)	0.19 (0.09)	0.382	0.265	0.77
8(9)-EpETrE	0.61 (0.22)	0.6 (0.19)	0.48 (0.17)	0.52 (0.15)	0.32 (0.14)	0.43 (0.13)	0.421	0.946	0.197
5(6)-EpETrE	0.53 (0.21)	1 (0.34)	0.63 (0.26)	0.89 (0.3)	0.49 (0.18)	0.69 (0.21)	0.475	0.614	0.103
14,15-DiHETrE	4.45 (0.33)	4.55 (0.57)	4.23 (0.37)	4.41 (0.46)	4.23 (0.37)	3.91 (0.2)	0.494	0.249	0.995
11,12-DiHETrE	3.77 (0.21)	4.03 (0.36)	3.6 (0.27)	4 (0.47)	3.85 (0.4)	3.61 (0.29)	0.834	0.219	0.721
8,9-DiHETrE	1.59 (0.17)	1.57 (0.18)	1.51 (0.15)	1.48 (0.16)	1.67 (0.21)	1.55 (0.09)	0.703	0.514	0.832
5,6-DiHETrE	3.43 (0.75)	4.12 (0.95)	3.91 (0.87)	2.52 (0.39)	3.28 (0.38)	4.03 (0.66)	0.69	0.565	0.312

Supplementary Table 3. Lipid mediator metabolites from the n-6 PUFA AA in patient plasma. Data is expressed as mean (SEM) in pg/mL of plasma. P-values were derived from the mixed effects ANOVA containing intervention (Interv; n-PUFA vs. placebo) and progress (early and end samples) as a within-subjects effect and as a between subjects effect. Intervention:progress (Interv:Prog) represents the significance of the interaction between these two terms.
Abbreviations: HETE, Hydroxyeicosatetraenoic acid; KETE, Oxo-eicosatetraenoic acid; HHTrE, Hydroxyheptadecatrienoic acid; DiHETE, Dihydroxyeicosatetraenoic acid; EpETrE, Epoxyeicosatrienoic acid; DiHETrE, Dihydroxyeicoatrienoic acid

Supplementary Table 4: Plasma LA metabolites

	n-PUFA (n=9)			Placebo (n=12)			P-values		
	**Baseline**	**Early**	**End**	**Baseline**	**Early**	**End**	**Interv**	**Progress**	**Interv:Progr**
C18 LA & GLA Metabolomes									
9-HODE	74.4±10.9	95.5±20.4	60.3±8.95	81.9±13.3	77.1±17.2	72.5±8.15	0.858	0.099	0.199
13-HODE	100±10.6	112±18.2	83.0±12.9	116±18.4	103±21.0	104±11.1	0.776	0.256	0.212
9-KODE	11.5±2.16	11±2.7	10.1±3.65	16.8±3.54	15.4±3.95	16.0±3.69	0.287	0.952	0.757
13-KODE	27.2±4.65	24.3±4.0	18.5±2.82	26.6±3.39	25.8±3.63	28.5±3.73	0.218	0.565	0.126
9(10)-EpOME	12.7±1.37	13.0±2.19	10.4±1.81	17.2±2.79	14.0±1.98	14.9±1.79	0.222	0.628	0.334
12(13)-EpOME	26.0±2.95	32.4±8.55	20.7±3.75	33.0±5.20	27.0±5.24	29.8±4.55	0.767	0.404	0.183
9,10-DiHOME	43.1±11.0	59.3±17.0	31.6±8.24	48.2±10.5	52.0±14.3	64.9±9.44	0.36	0.534	0.099
12,13-DiHOME	33.1±5.25	41.8±10.9	25.0±4.39	32.5±5.94	30.8±6.8	34.0±7.99	0.904	0.36	0.187
9,10,13-TriHOME	3.29±0.33	4.62±1.05	3.11±0.35	2.81±0.37	2.76±0.26	3.33±0.35	0.2	0.309	0.031
9,12,13-TriHOME	5.96±2.55	9.50±6.07	1.65±0.37	3.40±1.38	1.70±0.36	9.69±8.29	0.982	0.991	0.169
EKODE	151±31.8	104±25.1	123±36.1	160±40.1	137±34.3	132±40.0	0.675	0.636	0.415
13-HOTrEγ	1.35±0.35	0.9±0.17	0.69±0.11	0.6±0.13	0.74±0.18	0.92±0.28	0.901	0.912	0.145
C20 DGLA and EDE Metabolomes									
15-HETrE	1.43±0.18	1.09±0.12	0.91±0.09	1.28±0.13	1.68±0.27	1.61±0.31	0.065	0.325	0.655
8-HETrE	1.79±0.24	1.67±0.31	1.66±0.24	1.76±0.14	2.14±0.18	1.61±0.17	0.447	0.108	0.119
5-HETrE	2.05±0.3	1.88±0.48	1.26±0.22	1.66±0.27	1.79±0.19	1.44±0.18	0.883	0.054	0.559
PGE_1	0.21±0.07	0.12±0.04	0.2±0.05	0.15±0.04	0.15±0.06	0.17±0.05	0.945	0.013	0.174
PGD_1	0.02±0	0.02±0	0.02±0	0.04±0.02	0.06±0.02	0.05±0.02	0.223	0.4	0.4
TXB_1	0.82±0.26	1.2±0.46	0.68±0.35	2.2±0.85	1.56±0.57	1.38±0.58	0.477	0.094	0.419
11-HEDE	0.66±0.19	0.68±0.29	0.55±0.2	0.75±0.19	0.73±0.27	0.73±0.21	0.709	0.709	0.704

Supplementary Table 4: Plasma LA metabolites

	n-PUFA (n=9)				Placebo (n=12)				P-values		
C18 ALA Metabolome											
13-HOTrE	11.9±1.38	13.8±2.38	9.18±1.15		11.1±1.95	9.33±1.38	12.1±1.29		0.661	0.547	0.021
9-HOTrE	6.43±0.87	8.82±2.22	4.92±0.68		6.07±1.1	5.33±0.99	5.63±0.66		0.283	0.142	0.089
9-KOTrE	0.94±0.14	0.99±0.17	0.85±0.27		1.18±0.16	0.97±0.19	1.11±0.17		0.634	0.98	0.315
15(16)-EpODE	10504.9±1283.89	16673.39±4851.33	7657.52±1367.92		10624.25±1948.48	10771.8±4278.08	11996.31±1757.29		0.819	0.284	0.164
12(13)-EpODE	1.08±0.17	2.01±0.89	0.83±0.22		1.27±0.22	0.92±0.18	1.16±0.17		0.378	0.273	0.104
9(10)-EpODE	0.02±0	0.02±0	0.02±0		8.32±8.3	0.02±0	25.9±15.1		0.158	0.158	0.158

Supplementary Table 4. Lipid mediator metabolites from the n-6 PUFA LA in patient plasma. Data is expressed as mean (SEM) in pg/mL of plasma. P-values were derived from the mixed effects ANOVA containing intervention (Interv; n-PUFA vs. placebo) and progress (early and end samples) as a within-subjects effect and as a between subjects effect. Intervention:progress (Interv:Prog) represents the significance of the interaction between these two terms.

Supplementary Table 5. Additional compounds

Compound	Parent (M-1; *m/z*)	Product (M-1; *m/z*)	CE (eV)	Cone V
16,17-EpDPA	343.0	188.8	14	30
7,8-EpDPA	343.0	188.8	12	30
14,15-EpETE	317.0	174.7	12	30
16-17-DiHDPA	361.0	188.8	18	30
7,8-DiHDPA	361.0	188.8	16	30
8,9-EpETE	317.0	160.7	12	30
11,12-EpETE	317.0	166.7	12	30
10,11-EpDPA	343.0	152.6	12	30
13,14-EpDPA	343.0	152.6	16	30
11,12-DiHETE	335.0	166.7	14	30
10-11-DiHDPA	361.0	182.7	16	30
13-14-DiHDPA	361.0	188.8	16	30

Supplementary Table 5: Mass spectrometry parameters for the additional compounds added to the eicosanoid method reported in *Anal Chem* 2018; 90: 10239-10248

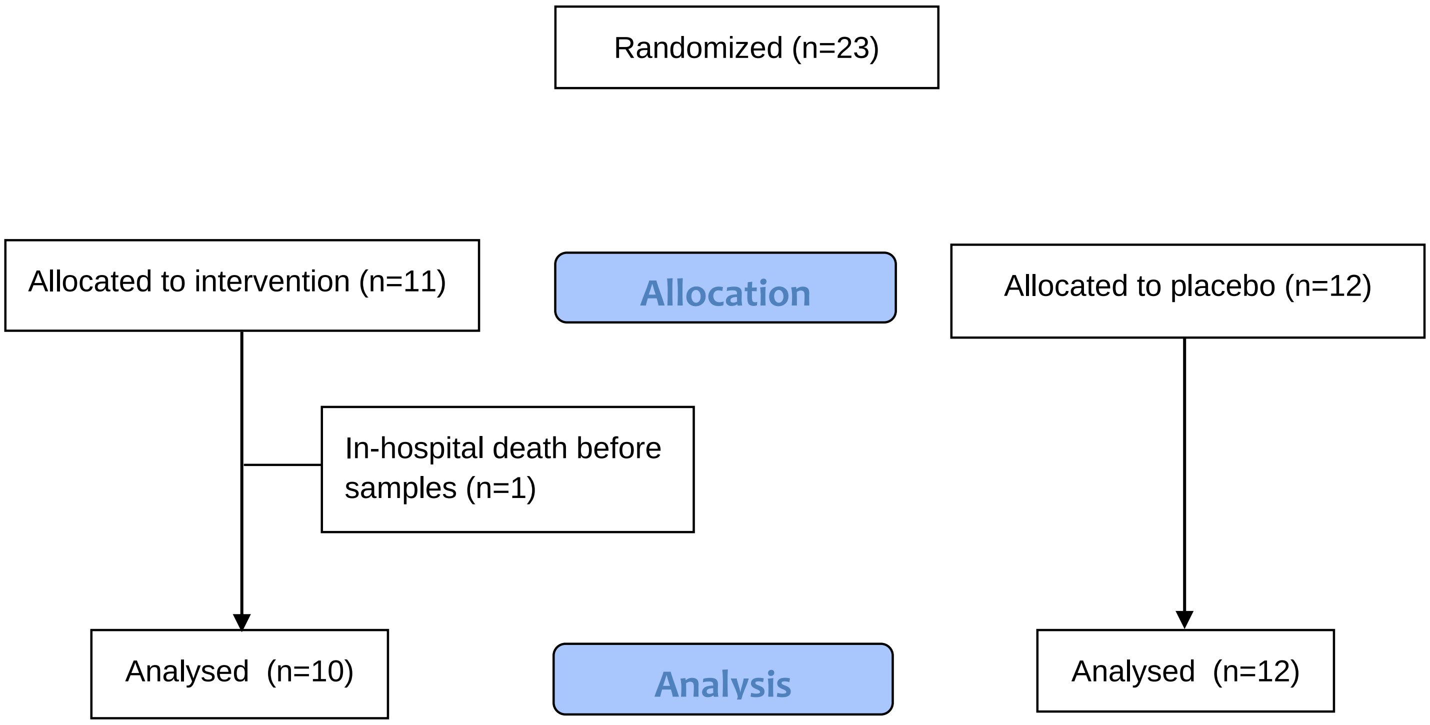

Supplementary Figure 1: Flow chart for the allocation and analysis in the trial.

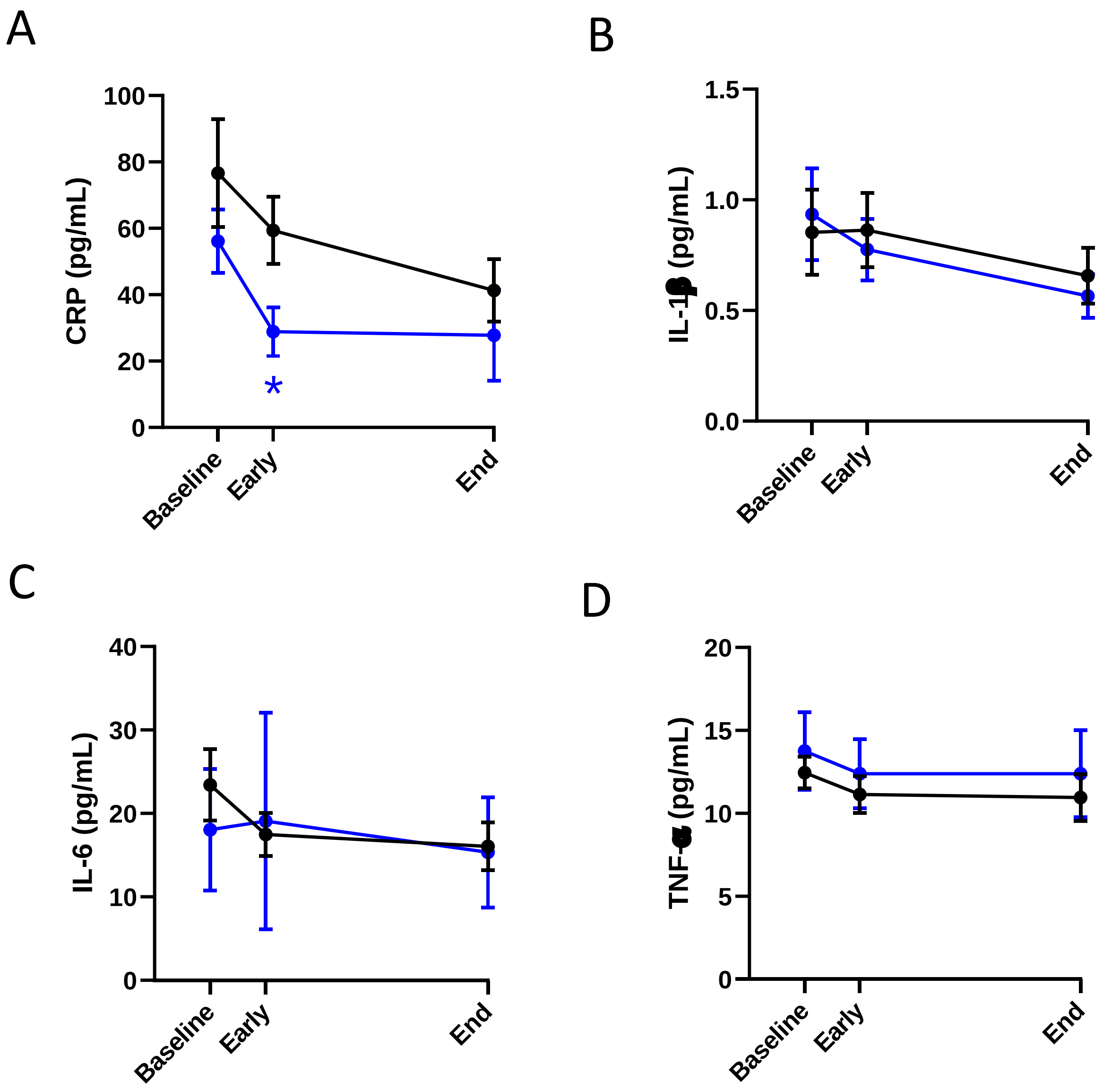

Supplementary Figure 2: (A) CRP (B) IL-1β, (C) IL-6 and (D) TNF-α levels (pg/mL) in patient plasma. CRP and cytokines were measured in plasma collected from patients at baseline, 48 h (Early) and after treatment (End) with intravenous infusion (2 mL/kg) of either placebo (NaCl; black n=12) or n-3 PUFA emulsion containing 10 g of fish oil per 100 mL (blue n=10). Results are expressed as mean ± SEM. * $p<0.05$ for n-PUFA compared with placebo (2-way ANOVA).

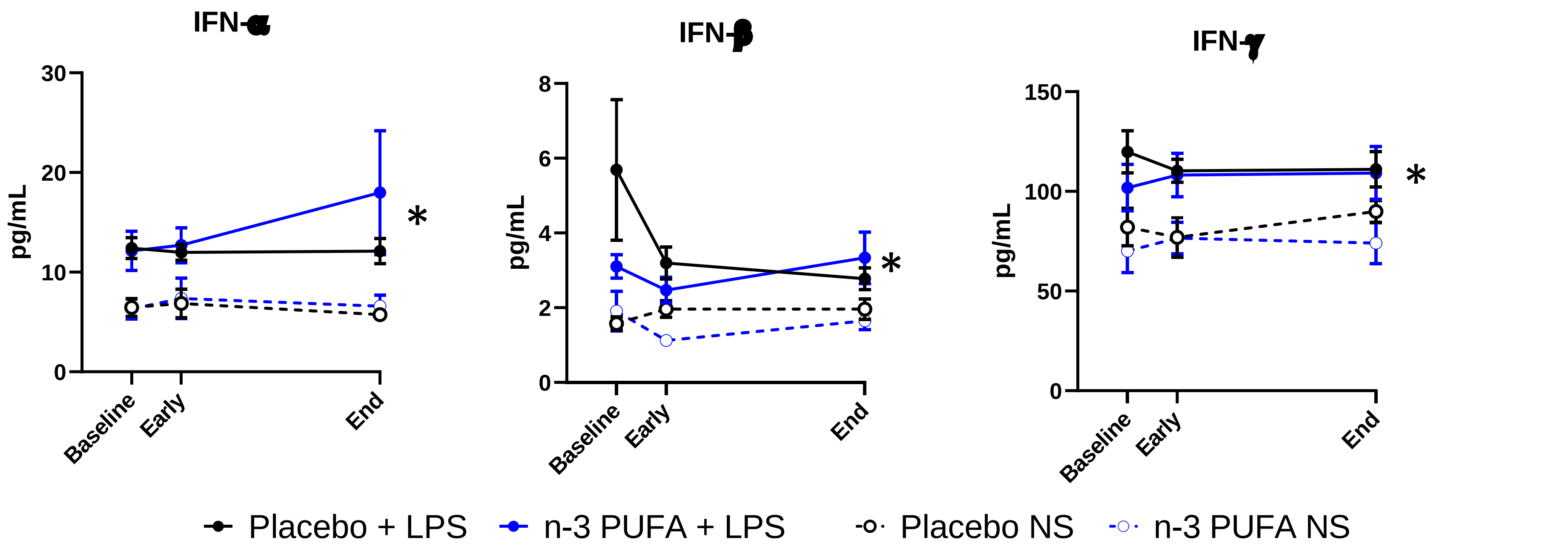

Supplementary Figure 3. Interferon (IFN) α, β, and γ in supernatants from non-stimulated (NS, dotted lines) and, LPS-stimulated (LPS, solid lines) PBMC isolated from patients at basline, at 48 h (Early), and after treatment (End) with intravenous infusion (2 mL/kg) of either placebo (black symbols NaCl); n=12) or n-3 PUFA emulsion containing 10 g of fish oil per 100 mL (blue symbols, LPS n=10; NS n=9) for 5 days. Results are expressed as mean ± SEM. * $p<0.05$ for LPS compared with non-stimulated (2-way ANOVA).

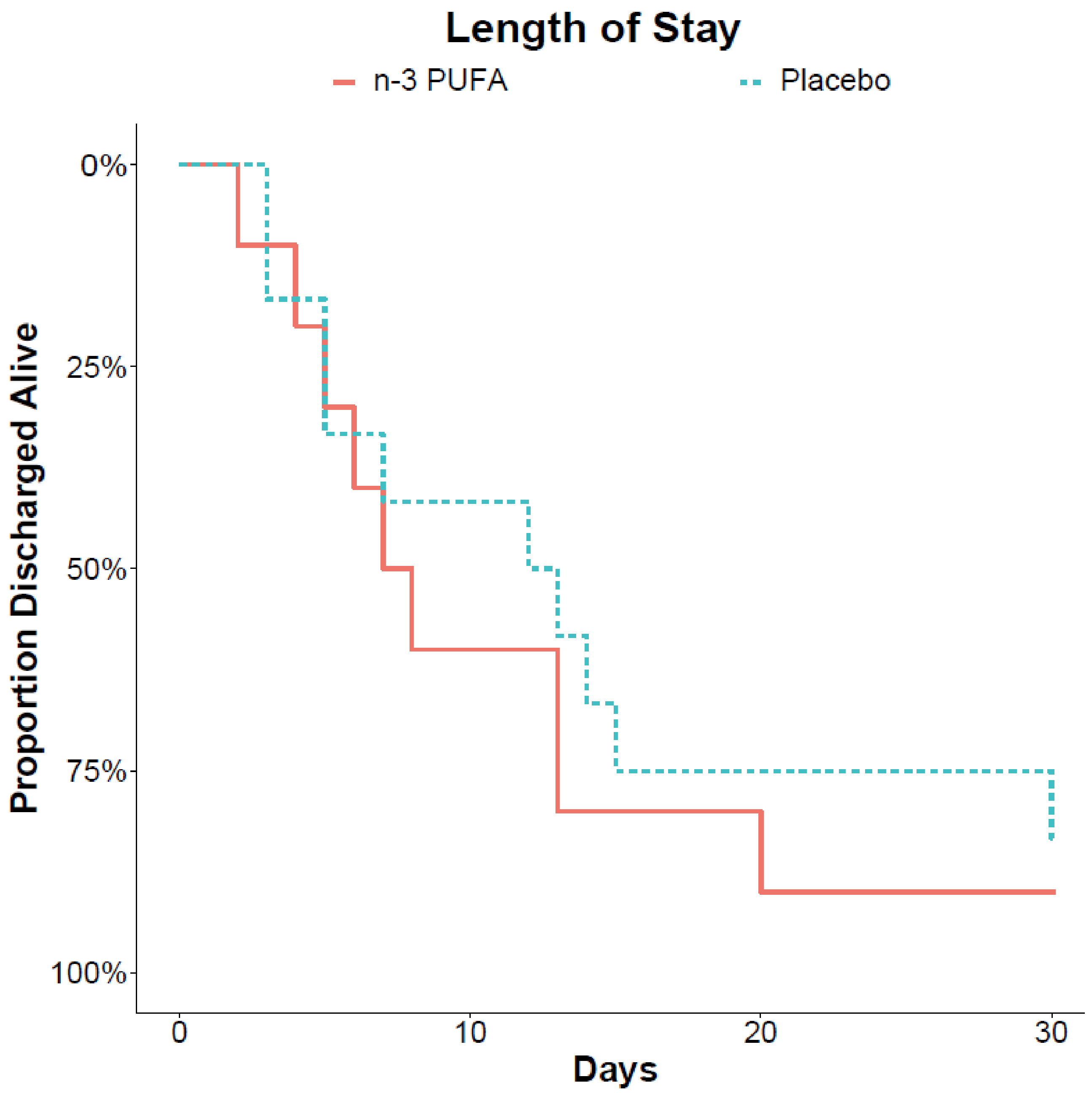

Supplementary Figure 4: Length of hospital stay after start of treatment with intravenous infusion (2 mL/kg) of either placebo (NaCl; dotted blue line n=12) or n-3 PUFA emulsion containing 10 g of fish oil per 100 mL (solid red line n=10). In-hospital deaths (for which 1 of in total 4 was excluded in this analysis as indicated in Supplementary Fig 1) were assigned the worst outcome, *e.g.* beyond 30 days, which was the longest recorded length of hospital stay for the subjects included in the analysis.
